# Impact of *IL10*, *MTP*, *SOD2*, and *APOE* Gene Polymorphisms on the Severity of Liver Fibrosis Induced by HCV Genotype 4

**DOI:** 10.3390/v13040714

**Published:** 2021-04-20

**Authors:** Amr Ali Hemeda, Amal Ahmad Mohamed, Ramy Karam Aziz, Mohamed S. Abdel-Hakeem, Marwa Ali-Tammam

**Affiliations:** 1 Department of Microbiology and Immunology, Faculty of Pharmaceutical Sciences and Pharmaceutical Industries, Future University in Egypt, Cairo 11835, Egypt; amr.ali@fue.edu.eg; 2 Department of Biochemistry and Molecular Biology, National Hepatology and Tropical Medicine Research Institute, Cairo 11562, Egypt; amalahmedhc@yahoo.com; 3 Department of Microbiology and Immunology, Faculty of Pharmacy, Cairo University, Cairo 11562, Egypt; ramy.aziz@gmail.com (R.K.A.); mohamed.salaheldin@pharma.cu.edu.eg (M.S.A.-H.)

**Keywords:** liver, hepatitis, HCV genotype 4, genetic variations, severity, haplotype

## Abstract

Complications of hepatitis C virus (HCV) chronic infection cause ~400,000 deaths worldwide annually. One complication, liver fibrosis, is influenced by host genetic factors. Genes influencing fibrosis include immune, metabolic, oxidative stress, and viral entry genes, such as interleukin 10 (*IL10*), microsomal triglyceride-transfer protein (*MTP*), superoxide dismutase-2 (*SOD2*), and apolipoprotein E (*APOE*)-encoding genes, respectively. Thus, correlating variations in these genes with HCV-induced fibrosis represents an attractive biomarker for the prognosis of fibrosis severity in chronically infected patients. Here, we aimed to test whether polymorphisms in *IL10*, *MTP*, *SOD2*, and *APOE* genes correlated with the severity of fibrosis induced by HCV genotype 4 (HCV-gt4) in a cohort of chronically infected Egyptian patients. Our results demonstrate a significant association between the severity of fibrosis and specific SNPs in IL-10, SOD2, and ApoE-encoding genes. Haplotype-combination analysis for *IL10*, *MTP*, *SOD2*, and *APOE* showed statistically significant associations between specific haplotype combinations and fibrosis severity. Identifying biomarkers correlating with the severity of HCV-gt4-induced fibrosis would significantly impact precision prophylaxis and treatment of patients at risk.

## 1. Introduction

Hepatitis C is a debilitating viral disease that annually claims the lives of about half a million humans worldwide [1]. Egypt is the country with the highest prevalence of hepatitis C in the world, with an estimated 14.8% of the population carrying the virus [2]. HCV is classified into seven main genotypes (1 to 7) that differ in their sequences by up to 30%. The most prevalent genotype in Egypt is genotype 4 [3]. HCV causes chronic disease in 70% of infected humans [2]. Chronic HCV has serious complications, including liver fibrosis, cirrhosis, and hepatocellular carcinoma (HCC) [4].

Liver fibrosis results from excessive connective tissue build-up in the liver (fibrogenesis) and can gradually lead to scarring of liver tissue and severe liver dysfunction. The influence of host genetic factors on the progression of liver fibrosis has recently become appreciated, arguing for the use of genetic markers to identify subjects at risk of rapid progression of fibrosis and developing severe disease. A wide array of host factors has been proposed to affect the rate of fibrosis progression and predispose patients with chronic hepatitis C virus (HCV) infection to the rapid progression of liver fibrosis [5]. These genes are suggested to play a substantial role in modulating the infection outcome and fibrosis process since single nucleotide polymorphisms (SNPs) within their gene loci correlate with the progression of liver fibrosis [6].

Gene polymorphisms influencing the course of liver diseases include several that were mapped to genes associated with the immune response, such as the gene encoding interleukin 10 (*IL10*) [5], and genes related to metabolism, such as the gene encoding microsomal triglyceride-transfer protein (*MTP*) [7], among other genes related to different physiological processes [8]. 

IL-10 has antifibrotic properties by shifting the intrahepatic immunological balance away from the predominance of Th1 cytokine [9], thereby exerting an anti-inflammatory impact [10]. IL-10 production differs between individuals, and this difference correlates with certain SNPs within the encoding gene and the associated regulatory regions [11]. Determination of *IL10* polymorphism may be crucial to predicting the probability of disease incidence and progression [12].

MTP is present in high concentration on the luminal side of the endoplasmic reticulum (ER) in the liver [13,14,15]. The prevalence of liver steatosis in HCV patients is significantly higher than in patients with other forms of chronic liver disease, such as hepatitis B or autoimmune hepatitis, suggesting a direct effect of HCV replication in the development of excess fat accumulation in the liver [16]. HCV may directly affect genes involved in lipid metabolism leading to fat accumulation in the liver [16].

Superoxide dismutases (SODs) are the first and most important line of antioxidant enzyme defense systems against reactive oxygen species (ROS) [17]. Chronic HCV infection showed a significantly increased SOD2 expression in peripheral blood mononuclear cells (PBMCs) compared to patients who resolved the infection [18]. The SOD2 targeting signal sequence polymorphism could affect the transport of the enzyme through the mitochondrial membrane, and a defect may alter the membrane receptor recognition site resulting in less of the enzyme protein entering the cell, thus lowering the antioxidant response to oxidative stress.

Apolipoprotein E (ApoE) is an essential element in the production of infectious HCV particles. ApoE is hijacked by the virus during HCV infection. ApoE is a key molecule required for HCV entry and is one of the possible therapeutic targets for interrupting HCV infection [19].

Although a number of studies have explored the impact of various human genetic polymorphisms on HCV-induced fibrosis, studies related to genotype 4a, the prevalent genotype in Egypt, are sparse.

Our study aimed to test whether polymorphisms in interleukin 10 (*IL10*), microsomal triglyceride transfer protein (*MTP*), superoxide dismutase 2 (*SOD2*), and Apolipoprotein E (*APOE*) genes are associated with fibrosis severity in patients chronically infected with HCV genotype 4. This would enhance our knowledge of how the interplay between host genetic factors and HCV sequence impacts the progression of liver fibrosis. Such knowledge will have a prognostic value and may potentially identify host factors that represent therapeutic targets for the treatment of fibrosis.

## 2. Materials and Methods

### 2.1. Ethical Approval

The study was approved by the Ethical Committee at the Faculty of Pharmacy, Cairo University, with ethical approval number MI (2039). Written informed consent was obtained from all patients.

### 2.2. Patients

The study was conducted on 100 adult Egyptian patients with chronic HCV genotype 4 (HCV-gt4) infection. The 100 patients included 34 female and 66 male patients aged 20–60 years. The blood samples were collected from patients diagnosed at the National Hepatology and Tropical Medicine Research Institute (Cairo, Egypt) between August and December 2017. 

The 100 HCV-4 positive patients were classified into two groups, according to the stage of liver fibrosis identified by liver biopsy and Fibroscan (scored by Ishak’s scoring system [20]). No discrepancy was observed between the staging results obtained by liver biopsy and Fibroscan. Group 1 included patients with mild fibrosis (from F1–F3), and group 2 included patients with severe fibrosis (From F4–F6).

Enrolled patients had chronic HCV infection (more than 6-month’s duration), HCV RNA positive, HBsAg negative, elevated serum aminotransferases, and were not previously treated with any antiviral drugs. Those suffering from decompensated liver cirrhosis, autoimmune liver disease, chronic renal failure, known thyroid disease or abnormal baseline thyroid-stimulating hormone (TSH) level, uncontrolled diabetes mellitus, or hypertension, as well as alcoholics, were excluded.

The provided demographic and clinical data included age, gender, Albumin, alpha-fetoprotein (AFP), thyroid-stimulating hormone (TSH), alkaline phosphatase (ALP), aspartate transaminase (AST), alanine transaminase (ALT), total bilirubin, direct bilirubin, glucose level, hemoglobin (Hb), creatinine, platelet count, white blood cell (WBC) count (Supplementary Tables S1 and S2) [21].

### 2.3. DNA Extraction and Analysis of IL-10, MTP, SOD2, and ApoE Polymorphism

DNA was extracted from whole blood samples with the QIAamp^®^ DNA blood mini kit (Qiagen^®^, Valencia, CA, USA) according to the manufacturer’s instructions.

**Figure 1:**
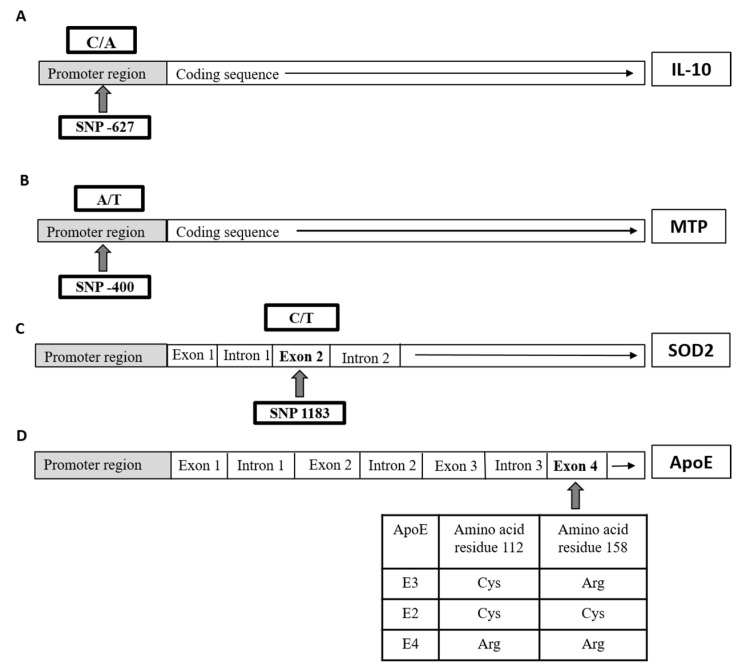
Schematic illustration of each single nucleotide polymorphisms (SNP) location on the gene level of (**A**) interleukin 10 (*IL10*), (**B**) microsomal triglyceride-transfer protein (*MTP*), (**C**) superoxide dismutase-2 (*SOD2*), and (**D**) apolipoprotein E (*APOE*) in the studied groups.

The polymerase chain reaction-restriction fragment length polymorphism (PCR-RFLP) was used to identify polymorphisms of *IL10* gene, at position-627; *MTP* gene, at position-400; *SOD2* gene, at position 1183; and *ApoE* gene as previously described [6,22,23,24], respectively (Figure 1), with slight modifications. The sequences of used primers as well as PCR products of all four genes are listed in Table 1.

The PCR products of all four genes were digested by different restriction enzymes (New England Biolab^®^, UK) (Table 1). The digestion pattern of each reaction of the four genes was developed after the products were run on 3%, 1.5%, 3.5%, and 3% agarose gels, respectively. Restriction digestion products are listed in Table 1 according to the genotype obtained for each gene.

### 2.4. Statistical Analysis

The significance of differences in genotype and allele frequencies between the Mild and Severe fibrosis groups was estimated by the Chi-square (χ2) test. The exact test was used instead when the expected frequency was <5. Odds ratios (OR) with 95% confidence intervals were calculated. For interpretation of results, test results with *p*-values < 0.05 suggested statistically significant differences.

Univariate and multiple binary logistic regression analyses were completed to detect predictors associated with the risk of severe fibrosis. The significant data in the univariate analysis were further analyzed by multiple analyses to determine the independent variables that affected the fibrosis severity. 

SPSS Statistics v. 20 for Windows (SPSS, Chicago, IL, USA) was used for statistical analyses, and GraphPad Prism 7 (GraphPad Inc., San Diego, CA, USA) was used for plotting graphs.

## 3. Results

### 3.1. Genotyping of IL-10 Encoding Gene Polymorphism

The IL-10-encoding gene has two alleles at position −627(C/A, rs1800872), leading to three genotypes: CC (homozygous wild type), CA (heterozygous mutant), and AA (homozygous mutant).

**Figure 2:**
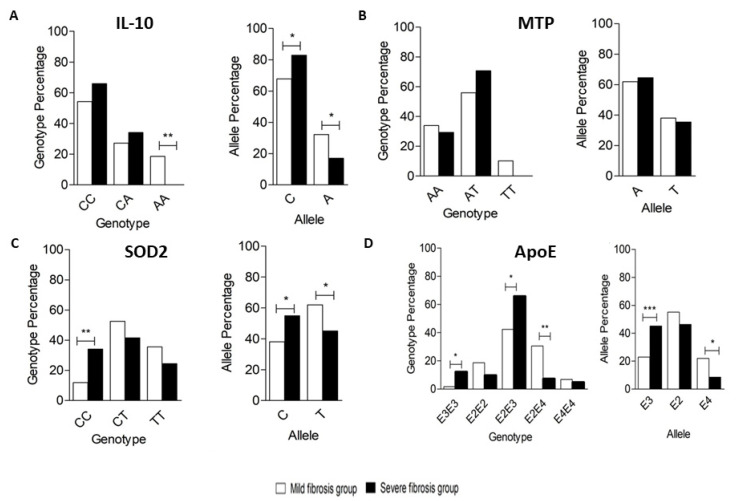
Genotyping and allele frequency of (**A**) *IL10*, (**B**) *MTP*, (**C**) *SOD2*, and (**D**) *APOE* in the studied groups. (**A**) Genotypes for the -627 *IL10* polymorphisms in the Mild and Severe groups showing the frequency of distribution of each genotype in both groups by percentage (left panel); Alleles for the -627 *IL10* polymorphisms in the Mild and Severe groups showing the frequency of distribution of each allele in both groups by percentage (right panel). (**B**) Genotypes for the -400 *MTP* (left panel); Alleles for the -400 *MTP* (right panel). (**C**) Genotypes for the 1183 *SOD2* (left panel); Alleles for the 1183 *SOD2* (right panel). (**D**) Genotypes for *APOE* (left panel), Alleles for *APOE* (right panel). * *p* < 0.05, ** *p* < 0.01, and *** *p* < 0.001.

Among 100 tested subjects, the most common genotype was CC (59%), followed by CA (30%), and then AA (11%). In the Mild group, the frequency of CC, CA, and AA genotypes was 54.2%, 27.1%, and 18.6%, respectively, while the frequency of CC, CA, and AA genotypes in the Severe group was 65.9%, 34.1%, and 0%, respectively. A significant difference was only observed in the frequency of genotype AA between both groups (*p*-value < 0.05). In the Mild group, the frequency of the C allele and A allele was 67.8% and 32.2%, respectively, while, in the Severe group, the frequency of the C allele and A allele was 82.9% and 17.1%, respectively. A significant difference in frequency was observed between the C and A alleles in both groups (*p*-value < 0.05) (Figure 2A).

To investigate the association between *IL10* (genotypes and alleles) and the susceptibility for development of severe fibrosis between the Mild and Severe groups, we calculated the OR for the risk of severe fibrosis development, using the CC genotype and C allele as references for genotype and allele, respectively (Table 2). The analysis showed no significant effect of any *IL10* genotypes on the development of severe fibrosis in both groups while comparing the allele distribution indicated that the low risk of developing severe fibrosis (protective effect) was associated with A allele (*p*-value = 0.016, OR 0.433 (95% C.I. 0.22–0.87) in both groups.

### 3.2. Genotyping of MTP-Encoding Gene Polymorphism 

Variations in the *MTP*-encoding gene at position -400(A/T, rs1491246) could be classified into three genotypes: AA (homozygous wild type), AT (heterozygous mutant), and TT (homozygous mutant).

Among 100 subjects, the most common genotype was AT, accounting for 62% of the subjects, followed by AA (32%) and then TT (6%). In the Mild group, the frequency of AA, AT, and TT genotypes was 33.9%, 55.9%, and 10.2%, respectively, while, in the Severe group, the frequency of AA, AT, and TT genotypes was 29.3%, 70.7%, and 0%, respectively. No significant genotype frequency difference was observed between both groups (*p*-value > 0.05). In the Mild group, the frequency of the A and T alleles was 61.9% and 38.1, respectively, while the frequency of the A and T alleles in the Severe group was 64.6% and 35.4%, respectively. No significant difference was observed in frequency between A and T alleles in either group at a *p*-value threshold of 0.05 (Figure 2B).

To investigate the association between *MTP* (genotypes and alleles) and the susceptibility of severe fibrosis development between Mild and Severe fibrosis groups, we calculated the OR for the risk of severe fibrosis development, using the AA genotype and A allele as references for genotype and allele, respectively (Table 2). The analysis showed no significant difference in the effect of any genotypes and alleles of MTP with the development of severe fibrosis in both groups.

### 3.3. Genotyping of SOD2-Encoding Gene Polymorphism

The *SOD2*-encoding gene has three genotypes at position 1183 (C/T, rs4880): CC (homozygous wild type), CT (heterozygous mutant), and TT (homozygous mutant) among our cohort.

Among 100 subjects, the most common genotype was CT, representing 48% of the subjects, followed by TT (31%) and CC (21%). In the Mild group, the frequency of CC, CT, and TT genotypes was 11.9%, 52.5%, and 35.6%, respectively, while in the Severe group, the frequency of CC, CT, and TT genotypes was 34.1%, 41.5%, and 24.4%, respectively. A significant difference was observed in the frequency of CC genotypes between both groups at *p*-value < 0.05. In the Mild group, the frequency of the C and T alleles was 38.1% and 61.9%, respectively, while the frequency of the C and T alleles was 54.9% and 45.1%, respectively, in the Severe group. A significant difference was observed in frequency between C and T alleles in both groups at *p*-value < 0.05 (Figure 2C).

To explore the association between *SOD2* (genotypes and alleles) and the susceptibility of severe fibrosis development between Mild and Severe fibrosis groups, we analyzed the OR for the risk of severe fibrosis development, using the CC genotype and C allele as references for genotype and allele, respectively (Table 2). The analysis showed that the lowest risk for developing severe fibrosis was associated with the TT genotype (*p*-value = 0.015, OR 0.238 (95% C.I. 0.07–0.77)), when compared with AT genotype (*p*-value = 0.016, OR 0.274 (95% C.I. 0.09–0.81)). On the other hand, comparing the allele distribution indicated that the low risk of developing severe fibrosis was associated with the T allele (*p*-value = 0.019, OR 0.507 (95% C.I. 0.29–0.90)) in both groups.

### 3.4. Genotyping of ApoE-Encoding Gene Polymorphism 

The ApoE-encoding gene has six genotypes: E3E3 (homozygous wild type), E3E2, E2E4, and E4E3 (heterozygous mutant), and E2E2 and E4E4 (homozygous mutant) among our cohort. There are two RefSeq (rs) defined for the *APOE* gene (rs429358 and rs7412).

Among 100 subjects, the most common genotype was E2E3 (52% of the subjects), followed by E2E4 (21%), and then E2E2, E3E3, and E4E4 representing 15%, 6%, and 6% of the subjects, respectively. The E4E3 genotype was not detected in any of our subjects. In the Mild fibrosis group, the frequency of E3E3, E2E2, E2E3, E2E4, and E4E4 genotypes were 1.7%, 18.6%, 42.4%, 30.5%, and 6.8%, respectively, while, in the severe fibrosis group, the frequency of E3E3, E2E2, E2E3, E2E4, and E4E4 genotypes were 12.2%, 9.8%, 65.9%, 7.3%, and 4.9%, respectively. A significant difference was observed in the frequency of E3E3, E2E2, and E2E4 genotypes between both groups (*p*-value < 0.05). In the Mild fibrosis group, the frequency of the E3, E2, and E4 alleles was 22.9%, 55.1%, and 22%, respectively, while the frequency of the E3, E2, and E4 alleles was 45.1%, 46.3%, and 8.5%, respectively, in the Severe fibrosis group. A significant difference was observed in frequency between E3 and E4 alleles in both groups at *p*-value < 0.05 (Figure 2D).

To explore the association between *APOE* (genotypes and alleles) and the susceptibility of severe fibrosis development between the Mild and Severe fibrosis groups, we statistically analyzed the OR for the risk of severe fibrosis development, using the E3E3 genotype and E3 allele as references for genotype and allele, respectively (Table 2). The analysis showed that the lowest risk for developing severe fibrosis was associated with E2E4 genotype (*p*-value = 0.004, OR 0.033 (95% C.I. 0.002–0.39)) compared with E2E2 genotype (*p*-value = 0.046, OR 0.072 (95% C.I. 0.006–0.829)). On the other hand, comparing the allele distribution, the lowest risk of developing severe fibrosis (protective effect) was associated with the E4 allele (*p*-value = 0.001, OR 0.197 (95% C.I. 0.074–0.591)), compared with E2 allele (*p*-value = 0.009, OR 0.427 (95% C.I. 0.226–0.807)) in both groups.

Finally, to test whether the observed impact of any of the polymorphisms was significantly different between males and females, we statistically examined the relation between gender and the different studied polymorphism using the Chi-square test. No significant difference was found between males and females, except for *SOD2* T/T genotype (was more frequent in females, *p*-value = 0.0052) and *APOE* E2/E3 genotype, which was more frequent in males (*p*-value = 0.0172).

### 3.5. Haplotype Analysis

#### 3.5.1. Four Genes Haplotype Analysis 

We next sought a rather novel approach to statistical analysis of the different haplotype combinations generated from the genotyping of the four tested genes. Twenty-four haplotype combinations were generated from the four selected encoding genes (-627 *IL10*, -400 *MTP*, 1183 *SOD2*, and *APOE*) among Mild and Severe fibrosis groups. Each haplotype consisted of four alleles arranged in the order: *IL10* (C or A), *MTP* (T or A), *SOD2* (C or T), and *APOE* (E3 or E2 or E4), respectively.

Haplotype CATE2 (*p*-value = 0.006, OR 0.189 (95% C.I. 0.057–0.620)), AATE2 (*p*-value = 0.010, OR 0.055 (95% C.I. 0.006–0.495)), ATTE4 (*p*-value = 0.014, OR 0.063 (95% C.I. 0.007–0.574)), CTTE4 (*p*-value = 0.032, OR 0.147 (95% C.I. 0.025–0.844)), and CTTE2 (*p*-value = 0.035, OR 0.314 (95% C.I. 0.107–0.923)) showed significant association with a low risk of (protective effect from) developing severe fibrosis stages, while haplotypes with zero frequencies were excluded from analysis.

#### 3.5.2. Three and Two Gene Haplotype Analysis

After haplotype analysis, the interaction between three and two genes alternately resulted in different allele combinations, and the OR was calculated for each combination. For the three-gene combination, the combined effect of *IL10*, *SOD2*, and *APOE* (Table 3), and low risk of severe fibrosis stages (protective from severe fibrosis stages) was observed in the combined effect of the ATE4, ATE2, CTE2, and CTE4 alleles (Table 3)

The combined effect of *IL10*, *MTP*, and *SOD2* (Table 3) and low risk of severe fibrosis (development) (protective from severe fibrosis stages) was observed in the combined effect of the CAT, ATT, and AAT alleles.

The combined effect of *IL10*, *MTP*, and *APOE* (Table 3) and low risk of severe fibrosis stages (protective from severe fibrosis stages) was observed in the combined effect of the AAE2, ATE4, CAE2, and CTE4.

The combined effect of *MTP*, *SOD2*, and *APOE* (Table 3) and low risk of severe fibrosis stages (protective from severe fibrosis stages) was observed in the combined effect of the ATE2, TTE4, ACE2, ATE4, and TTE2 alleles.

As for two-gene combinations, the combined effect of *IL10* and *MTP* (Table 4) and low risk for severe fibrosis stages was observed in the combined effect of AA alleles).

The combined effect of *IL10* and *SOD2* (Table 4) and low risk for severe fibrosis stages was observed in the combined effect of the AT alleles and CT alleles.

The combined effect of *IL10* and *ApoE* (Table 4) and low risk for severe fibrosis stages was observed in the combined effect of the AE2 AE4, CE4, and CE2 alleles.

The combined effect of *MTP* and *SOD2* (Table 4) and low risk for severe fibrosis stages was observed in the combined effect of the AT alleles.

The combined effect of *MTP* and *ApoE* (Table 4) and low risk for severe fibrosis stages was observed in the combined effect of the AE2, TE4, and AE4 alleles.

The combined effect of *SOD2* and *ApoE* (Table 4) and low risk for severe fibrosis stages was observed in the combined effect of the TE4 and TE2 alleles.

## 4. Discussion

Like many diseases, hepatic fibrosis is influenced by multiple genes that interact with environmental factors [25]. Association between the development of liver fibrosis in patients with HCV and multiple SNPs influences fibrosis progression [5,26]. Numerous association studies have explored the role of gene polymorphisms in the development of liver fibrosis and/or cirrhosis in patients with various types of chronic liver disease. Specifically, genetic polymorphisms are associated with hepatic disease progression caused by different HCV genotypes [27]; yet studies related to genotype 4a (gt4a), the prevalent genotype in Egypt, are sparse. Since HCV prevalence in Egypt is the highest in the world, the current study was designed to identify polymorphisms in different encoding genes and test their association with the severity of fibrosis in Egyptian patients with HCV.

Indeed, significant associations were found in Egyptian patients with HCV between mild fibrosis stages and the A allele of the IL-10-encoding gene, at position -627 promoter region; the CT and TT genotypes of the SOD2-encoding gene, at position 1183 mitochondrial targeting sequence; the T allele (Valine) of the SOD2-encoding gene, at position 1183 mitochondrial targeting sequence; the E2E2 and E2E4 genotypes of the ApoE-encoding gene, and E2 and E4 alleles of the ApoE-encoding gene. These associations suggest the above genotypes/alleles provide protection from severe fibrosis stages.

IL-10 is an anti-inflammatory cytokine that plays an important role in the regulation of the immune response. As one of the many cytokines involved in the anti-HCV immune response, it shifts the Th1/Th2 balance by reducing the Th1 response and suppressing pro-inflammatory cytokines, such as TNFα and IFNγ secretion [28]. We investigated the significance of promoter polymorphism at -627 C/A of the IL-10-encoding gene and the liver fibrosis severity in a cohort of Egyptian patients chronically infected with HCV-gt4a and found no significant association between fibrosis severity and genotypes in the studied groups. Two other studies [22,29] investigated the effect of IL-10-encoding gene promoter polymorphism at the -627 promoter region on alcoholic liver disease, but not HCV, and found that the A allele in the *IL10* promoter region -627 is associated with an increased risk of advanced liver disease among heavy drinkers. The authors relied on functional evidence that correlates the -627 A allele with low IL-10 expression that favors inflammatory, immune-mediated, and profibrotic liver injury mechanisms.

The previously mentioned studies reported a controversial association between the *IL10* promoter gene polymorphism and disease progression, which were inconsistent with our results, perhaps because of differences in IL-10 homologs and different IL-10 binding receptors, which are likely to complicate the determination of levels of IL-10 expression in vitro [30]. The mechanism involved in most clinical liver pathologies is an unregulated inflammatory response, resulting in tissue damage, fibrosis, and cirrhosis [31]. Conflicting results between studies of association with polymorphism are not atypical, as they may be due to sample size differences, selection of subjects for study, genetic heterogeneity in different populations, and different gene–gene or gene–environment interactions [32].

MTP is a heterodimeric protein involved in triglyceride (TG), phospholipid, and cholesterol ester (CE) transport and lipoprotein assembly and catalysis [33]. Variations in this protein are associated with familial abetalipoproteinaemia [33,34], a notion supporting the hypothesis that HCV may trigger lipid accumulation in infected hepatocytes via direct interference with MTP, leading to very-low-density lipoprotein (VLDL)/TG retention [35]. Therefore, genetic polymorphisms in the *MTP*-encoding gene can modulate the concentration of MTP protein in the endoplasmic reticulum, which may have an impact on the secretion pattern of lipoprotein.

Here, we evaluated the association between the MTP-encoding gene promoter polymorphism at -400 A/T and fibrosis severity in Egyptian patients with HCV-gt4a, and, to the best of our knowledge, *MTP* polymorphism at this location has not been previously investigated in Egyptian patients. Having not found a significant association between *MTP* genotypes/alleles and HCV fibrosis severity in studied groups, we suggest that HCV-gt4a did not modulate *MTP* activity/transcription and *MTP* -400 A/T polymorphisms did not associate with the hepatic steatosis. Our results are in accordance with Karpe and colleagues’ conclusion [23] of no difference in transcriptional activity between constructs containing either of the two -400 A or T alleles. We propose the same explanation for our results.

In addition, patients with positive viremia and chronic HCV infection had a significantly increased expression of SOD2 in PBMCs relative to cases that had resolved the viremia [18]. We evaluated the association between the dimorphism in the *SOD2* mitochondrial targeting sequence at position 1183 C (encoding alanine) or T (encoding valine) and fibrosis severity in HCV gt4a Egyptian patients. We found that the presence of C/C (Ala/Ala) genotype was associated with HCV severe fibrosis, while the presence of C/T (Ala/Val) and T/T (Val/Val) genotypes were associated with HCV mild fibrosis. Moreover, the T was considered as a protective allele from HCV severe fibrosis compared with the C allele.

These results agree with Houldsworth et al. [36], who suggested that alanine, which leads to a 40% increased enzymatic activity due to the increase in SOD2 expression, may contribute to chronic inflammation in the HCV group by reducing oxidative stress and enhancing the environment for viral replication in the liver. This is supported by reports that HCV replication is enhanced by a decrease in the ROS level. However, the lower SOD2 response in the liver compared to the periphery and the lower SOD2-producing genotype (Val) may combine and produce a higher ROS environment resulting in poor viral replication. Further, Ezzikouri et al. [37] found that the C allele (Ala) *SOD2* variant was associated with a higher risk for HCC than in controls in a group of HCV-gt1 patients. Finally, Shimoda-Matsubayashi et al. [38] reported that the Ala-to-Val variation in the *SOD2* leader signal affects the enzyme’s processing efficiency. The valine form might be transferred to the mitochondria less effectively than the enzyme’s alanine form. Those same authors showed that basal *SOD2* activity for Ala/Ala may be strongest, followed by Ala/Val and then Val/Val [38].

In HCV, *ApoE* polymorphism was reported to predict various infection outcomes [39,40]. Here, we evaluated the association between *ApoE* polymorphisms and fibrosis severity in HCV gt4a Egyptian patients. Our results suggest that patients with E2/E2 and E2/E4 genotypes were protected from severe fibrosis stages compared with other genotypes. In addition, the E4 allele was considered the most protective allele from severe fibrosis stages. The possible mechanism for the action of *ApoE* E4 is that the replacement of arginine results in the so-called domain interaction [41]. This structural difference is thought to play a role in the altered function of *ApoE4* [42]. Another explanation is that the E4 allele is associated with increased serum levels of low-density lipoproteins (LDL), while HCV entry is through LDL-Rs [43].

Our findings are consistent with a recent study suggesting that the E4 allele, unlike E3 allele carriers, are protected against chronic HCV infection and have a slow progression of liver fibrosis [44]. In similar work by Gomaa et al. [45] on Egyptian patients with HCV who received HCV therapy, patients with the E4 allele were more likely to clear HCV infection and recover after combined therapy, while the E3 allele was considered as a risk factor for the chronicity of HCV and resistance to therapy. Moreover, Mueller et al. [46] reported that HCV-gt1 patients with the E4 allele were protected from severe HCV infection. Another study [24] investigated the role of *ApoE* polymorphism in modulating the progression of liver fibrosis in HCV-gt1b patients with normal transaminases and found that the E2 and E4 alleles were associated with less severe fibrosis in chronic HCV infection, unlike the E3 allele, which is considered as a risk factor for rapid fibrosis progression [24].

In addition, Hill et al. [47] and Price et al. [48] observed that HCV-gt1 and gt3 patients who carried the E2 and E4 alleles favored viral clearance, which influences HCV infection outcome. They assumed that the E2 allele, which poorly binds to its receptors, may be especially associated with decreased resistance to HCV infection by defective absorption of HCV lipoviroparticles (LVP) by the candidate receptors low-density lipoprotein – receptors (LDL-R) and scavenger receptor class B type 1 (SR-B1). Nevertheless, an earlier study about the effect of ApoE polymorphism and the outcome of HCV infection in the United States indicated that the E4 allele protected against severe liver disease in the HCV-gt1 American cohort [49].

By contrast, Stachowska et al. [50] evaluated Non-alcoholic fatty liver disease (NAFLD) and found that the E4 allele was significantly associated with advanced fibrosis. A previous study by Mueller et al. [51] investigated whether the presence of the E4 allele was associated with poor treatment response in HCV-gt1 German patients, while Lee et al. [52] found no association between *ApoE* genotypes/alleles and the pathogenesis of liver disease in Korean patients.

In addition, the study of the impact of the four studied genes polymorphism and gender showed that *SOD2* T/T genotype was more frequent in females and *APOE* E2/E3 genotype was more frequent in males. This has been evaluated in other disease conditions [53,54].

After having extensively analyzed each case of polymorphism independently, we assumed that performing the statistical analysis of the different haplotype combinations of the candidate SNPs in IL-10, MTP, SOD2, and ApoE-encoding genes could provide more significance in predicting the degree of HCV-gt4 induced liver fibrosis. This correlation between combined alleles for SNPs from multiple genes implicated in liver fibrosis represents a novel strategy that could be of significant prognostic value in predicting the degree of fibrosis.

To this end, we examined the statistical significance, which reflects the predictive value for the various haplotype combinations. When combining the different haplotypes for the tested SNPs in the four genes, *IL10*, *MTP*, *SOD2*, and *APOE*, we had 24 possible combinations. Of these, combined haplotypes CATE2, AATE2, ATTE4, CTTE4, and CTTE2 were associated with mild fibrosis with high significance *p*-value: 0.006, 0.010, 0.014, 0.032, and 0.035, respectively.

This strategy is beneficial in case of technical or economic inability to perform the PCR-RFLP for all four genes, as we showed that combined haplotype analysis for two or three genes could still provide a statistically significant prognostic value. The following combined haplotypes are associated with mild fibrosis: haplotype combinations ATE2 (*p*-value = 0.006) and ATE4 (*p*-value = 0.006) from the three genes *IL10*, *SOD2*, and *APOE*; haplotype combinations ATE2 (*p*-value = 0.002) and TTE4 (*p*-value = 0.005) from the three genes *MTP*, *SOD2*, and *ApoE*; haplotype combination AAE2 (*p*-value = 0.004) from the three genes *IL10*, *MTP*, and *APOE*.

Likewise, from examining the following pairs of genes, the combined haplotypes are associated with mild fibrosis: haplotype combinations AE2 (*p*-value = 0.007) and AE4 (*p*-value = 0.008) from the two genes *IL10* and *APOE*; haplotype combinations TE4 (*p*-value = 0.002) and TE2 (*p*-value = 0.005) from *SOD2* and *APOE*; haplotype combination TE4 (*p*-value = 0.004) from *MTP* and *APOE*; haplotype combination AT (*p*-value = 0.006) from *IL-10* and *SOD2*; and finally haplotype combination AA *(p*-value = 0.006) from *IL10* and *MTP*.

It is worth noting that the introduction of direct-acting antiviral agents (DAAs) has revolutionized the treatment of patients with chronic HCV; however, the majority of these patients are still at risk of progressing to liver complications, including fibrosis, cirrhosis, and hepatocellular carcinoma (HCC). These diseases are induced by insults to liver tissue following years of chronic HCV infection [55]. Various studies reported that, although DAAs are highly effective in treating patients with chronic hepatitis C, they do not eliminate the risk of progression to severe fibrosis, cirrhosis, and HCC [56,57]. 

Fibrosis represents the preceding step for all other liver complications, including cirrhosis and HCC. Thus, for optimal management of liver diseases in the age of precision medicine, it remains important to have clear prognostic markers for the progression of liver diseases in patients who suffered from chronic HCV, even if they have cleared the virus by DAA treatment.

On another front, although DAAs are highly effective, the treatment efficacy may be reduced in patients who have SNPs in certain genes [58]. Typing genes whose products may interfere with the action, disposition, and toxicity of DAAs, is thus an emerging pharmacogenetic and precision medicine research prospect. For example, *IL28B* genotyping can help in identifying patients that are more likely to have successful treatment in genotype 4 HCV patients [59].

In addition to genotyping, microbiome analysis is another tool for predicting prognosis and treatment outcomes in cases with chronic HCV [60,61]. We expect the future of managing and monitoring this debilitating disease to involve a combination of genotyping, pharmacogenomics, and microbiome analysis.

## 5. Conclusions

In this study, the analysis of Egyptian patients with HCV showed a significant association between the A allele of IL-10-encoding gene at position -627 promoter region, the T allele (Valine) of SOD2-encoding gene at position 1183 mitochondrial targeting sequence and the E2 and E4 alleles of ApoE-encoding gene and mild fibrosis stage. These polymorphisms are thus seen as protective alleles from severe fibrosis.

The haplotype combination analysis for IL-10, MTP, SOD2, and ApoE-encoding genes showed a statistically significant association between specific haplotypes and mild fibrosis stages. Accordingly, this study may direct the determination of the patient biomarker profile, which, in turn, defines who is at high risk of developing severe fibrosis and who is protected from severe fibrosis.

The current era is one of predictive personalized and preventive medicine. The continuous determination of genetic markers becomes crucial in therapeutic decisions prior to the onset of therapy for liver fibrosis in patients with chronic hepatitis C. Our results will contribute to the establishment of possible genetic markers for predicting the degree of fibrosis. This knowledge has a prognostic significance in patients chronically infected with HCV and may suggest a more aggressive therapy for those with increased risk of disease progression.

## Figures and Tables

**Table 1:** Primer sequences, enzymes used, and digested products obtained for detection of *IL-10*, *MTP*, *SOD2*, and *APOE* gene polymorphisms by polymerase chain reaction-restriction fragment length polymorphism (PCR-RFLP).

Gene	Oligonucleotide Sequence (5’-3’)	Product Size (bp)	Restriction Enzyme	Digested Products	Reference
***IL10*** **(-627)**	Forward: CCTAGGTCACAGTGACGTGG Reverse: GGTGAGCACTACCTGACTAGC	412	*RsaI*	**1** band of 412 bp for CC genotype **2** bands of 212 and 200 bp for **AA** genotype **3** bands of 412, 212, and 200 bp for **AC** genotype	[22]
***MTP*** **(-400)**	Forward: GTCCATACAAGAAAAATTAAAATTTGGTTAG Reverse: GTCCATACAAGAAATATTAAAATTTGGTTAG	838	*SspI*	**1** band of 838 bp for **AA** genotype **2** bands of 494 and 344 bp for **TT** genotype **3** bands of 838, 494, and 344 bp for **AT** genotype.	[23]
***SOD2*** **(1183)**	Forward: CTGACCGGGCTGTGCTTTCTCG Reverse: CTCCCGCCGCTCAGCCTGGACC	258	*BsaWI*	**1** band of 258 bp for **CC** genotype **2** bands of 210 and 48 bp for **AA** genotype **3** bands of 258, 210, and 48 bp for **CT** genotype.	[6]
***APOE***	Outer forward: TGAAGGAGTTGAAGGCCTAC	367	*HaeII & AflIII*	**3** bands of 145, 50, and 23 bp for **E3** allele **2** bands of 168 and 50 bp for **E2** allele **2** bands of 195 and 23 bp for **E4** allele	[24]
	Outer reverse: CACGCGGCCCTGTTCCACCA				
	
	Inner forward:				
	TCCAAGGAGCTGCAGGCGGCGCA	218			
	Inner reverse:				
	GCCCCGGCCTGGTACACTGCCA				

**Table 2:** Odds ratio (OR) for *IL-10*, *MTP*, *SOD2*, and *APOE* (genotypes and alleles) for both groups.

Gene Polymorphisms	Mild Group (N = 59)	Severe Group (N = 41)	OR	*p*-Value
	%	%		
***IL10* Polymorphism**				
Genotype				
CC	54.2	65.9	Ref.	
CA	27.1	34.1	1.037	0.936
AA	18.6	0.0	–	–
Allele				
C	67.8	82.9	Ref.	–
**A**	32.2	17.1	0.433	0.016 *
***MTP* Polymorphism**				
Genotype				
AA	33.9	29.3	Ref.	
AT	55.9	70.7	1.465	0.390
TT	10.2	0.0	–	–
Allele				
A	61.9	64.6	Ref.	–
T	38.1	35.4	0.888	0.690
***SOD2* Polymorphism**				
Genotype				
CC	11.9	34.1	Ref.	–
**CT**	52.5	41.5	0.274	0.016 *
**TT**	35.6	24.4	0.238	0.015 *
Allele				
C	38.1	54.9	Ref.	–
**T**	61.9	45.1	0.507	0.019 *
***APOE* Polymorphism**				
Genotype				
E3/E3	1.7	12.2	Ref.	–
**E2/E2**	18.6	9.8	0.072	0.046 *
E2/E3	42.4	65.9	0.216	0.209
**E2/E4**	30.5	7.3	0.033	0.004 **
E4/E4	6.8	4.9	0.100	0.242
Allele				
E3	22.9	45.1	Ref.	–
**E2**	55.1	46.3	0.427	0.009 **
**E4**	22.0	8.5	0.197	0.001 **

* *p* < 0.05, ** *p* < 0.01. Ref. = reference genotype or reference allele. All *p*-values < 0.05 are shown in bold.

**Table 3:** Analysis of three-gene combination alternately for alleles of *IL-10* -627 (C/A), *MTP* -400 (A/T), *SOD2* 1183 (C/T), and *APOE* (E3, E2, and E4) in Mild and Severe fibrosis groups.

Allele Combinations	Mild Group (N = 59)	Severe Group (N = 41)	*p*-Value	OR (95%CI)
	%	%		
***IL10* + *MTP* + *SOD2***				
CAC	21.2	42.7	Ref.	1.000
**CAT**	22.9	17.1	0.018 *	**0.370** (0.162–0.845)
CTC	6.8	6.1	0.199	0.446 (0.131–1.527)
CTT	16.9	17.1	0.112	0.500 (0.213–1.175)
AAC	7.6	0.0	–NA	–NA
**AAT**	10.2	4.9	0.024 *	**0.238** (0.069–0.825)
ATC	2.5	6.1	0.822	1.191 (0.260–5.447)
ATT	11.9	6.1	0.019 *	**0.255** (0.081–0.800)
***IL10*** **+ *MTP* + *APOE***				
CAE3	17.8	39.0	Ref.	1.000
**CAE2**	20.3	17.1	0.028 *	**0.383** (0.162–0.904)
CAE4	5.9	3.7	0.089	0.281 (0.065–1.21)
CTE3	2.5	2.4	0.387	0.438 (0.067–2.844)
CTE2	15.3	18.3	0.178	0.547 (0.227–1.317)
**CTE4**	5.9	2.4	0.049 *	**0.188** (0.035–0.991)
AAE3	2.5	2.4	0.387	0.438 (0.067–2.844)
**AAE2**	11.9	1.2	0.004 *	**0.047** (0.006–0.384)
AAE4	3.4	1.2	0.117	0.164 (0.017–1.571)
ATE3	0.0	1.2	–NA	–NA
ATE2	7.6	9.8	0.337	0.164 (0.194–1.753)
**ATE4**	6.8	1.2	0.023 *	**0.082** (0.010–0.705)
***IL10*** **+ *SOD2*+ *APOE***				
CCE3	11.9	30.5	Ref.	1.000
CCE2	11.9	15.9	0.200	0.520 (0.192–1.412)
CCE4	4.2	2.4	0.097	0.224 (0.038–1.309)
CTE3	8.5	11.0	0.228	0.504 (0.166–1.534)
**CTE2**	23.7	19.5	0.013 *	**0.320** (0.130–0.785)
**CTE4**	7.6	3.7	0.024 *	**0.187** (0.043–0.805)
ACE3	2.5	1.2	0.163	0.187 (0.018–1.969)
ACE2	6.8	4.9	0.068	0.280 (0.071–1.099)
ACE4	0.8	0.0	–NA	–NA
ATE3	0.0	2.4	–NA	–NA
**ATE2**	12.7	6.1	0.006 *	**0.187** (0.056–0.623)
**ATE4**	9.3	2.4	0.006 *	0.102 (0.020–0.526)
***MTP*** **+ *SOD2*+ *APOE***				
ACE3	11.9	30.5	Ref.	1.000
**ACE2**	13.6	9.8	0.020 *	**0.280** (0.096–0.818)
ACE4	3.4	2.4	0.170	0.280 (0.045–1.727)
ATE3	8.5	11.0	0.228	0.504 (0.166–1.534)
**ATE2**	18.6	8.5	0.002 *	**0.178** (0.061–0.521)
**ATE4**	5.9	2.4	0.035 *	**0.160** (0.029–0.878)
TCE3	2.5	1.2	0.163	0.187 (0.018–1.969)
TCE2	5.1	11.0	0.780	0.840 (0.247–2.853)
TCE4	1.7	0.0	–NA	–NA
TTE3	0.0	2.4	–NA	–NA
**TTE2**	17.8	17.1	0.040 *	**0.373** (0.146–0.957)
**TTE4**	11.0	3.7	0.005 *	**0.129** (0.031–0.532)

Ref. = reference allele. All * *p*-values < 0.05 are shown in bold. –NA: Not Applicable.

**Table 4:** Analysis of two-gene combination alternately for alleles of *IL-10* -627 (C/A), *MTP* -400 (A/T), *SOD2* 1183 (C/T), and *ApoE* (E3, E2, and E4) in the Mild and Severe fibrosis groups.

Allele Combinations	Mild Group (N = 59)	Severe Group (N = 41)	*p*-Value	OR (95%CI)
	%	%		
***IL10* + *MTP* Polymorphism**				
CA	44.1	59.8	Ref.	1.000
CT	23.7	23.2	0.359	0.720(0.357–1.452)
**AA**	17.8	4.9	0.006 *	**0.202**(0.065–0.631)
AT	14.4	12.2	0.290	0.624(0.261–1.495)
***IL10* + *SOD2* Polymorphism**				
CC	28.0	48.8	Ref.	1.000
**CT**	39.8	34.1	0.034 *	**0.491**(0.255–0.948)
AC	10.2	6.1	0.067	0.344(0.110–1.075)
**AT**	22.0	11.0	0.006 *	**0.286**(0.118–0.693)
***IL10* + *APOE* Polymorphism**				
CE3	20.3	41.5	Ref.	1.000
**CE2**	35.6	35.4	0.046 *	**0.487** (0.241–0.986)
**CE4**	11.9	6.1	0.019 *	**0.252** (0.080–0.794)
AE3	2.5	3.7	0.685	0.706 (0.131–3.801)
**AE2**	19.5	11.0	0.007 *	**0.276** (0.109–0.701)
**AE4**	10.2	2.4	0.008 *	**0.118** (0.024–0.574)
***MTP* + *SOD2* Polymorphism**				
AC	28.8	42.7	Ref.	1.000
**AT**	33.1	22.0	0.032 *	**0.448**(0.216–0.931)
TC	9.3	12.2	0.803	0.883(0.332–2.348)
TT	28.8	23.2	0.103	0.543(0.261–1.130)
***MTP* + *APOE* Polymorphism**				
AE3	20.3	41.5	Ref.	1.000
**AE2**	32.2	18.3	0.002 *	**0.279** (0.126–0.616)
**AE4**	9.3	4.9	0.034 *	**0.257** (0.073–0.903)
TE3	2.5	3.7	0.685	0.706 (0.131–3.801)
TE2	22.9	28.0	0.191	0.601 (0.280–1.290)
**TE4**	12.7	3.7	0.004 *	**0.141** (0.037–0.542)
***SOD2* + *APOE* Polymorphism**				
CE3	14.4	31.7	Ref.	1.000
CE2	18.6	20.7	0.128	0.505 (0.210–1.218)
CE4	5.1	2.4	0.081	0.218 (0.040–1.209)
TE3	8.5	13.4	0.539	0.719 (0.251–2.060)
**TE2**	36.4	25.6	0.005 *	**0.319** (0.143–0.713)
**TE4**	16.9	6.1	0.002 *	**0.164** (0.052–0.519)

Ref. = reference allele. All * *p*-values < 0.05 are shown in bold.

## Data Availability

The data presented in this study are available on request from the corresponding author.

## Supplementary Materials

The following are available online at https://www.mdpi.com/article/10.3390/v13040714/s1, Table S1: Patients’ demographic data [21]; Table S2: Patients’ clinical data [21].
