# Supplementary material for: Impact of IL10, MTP, SOD2, and APOE Gene Polymorphisms on the Severity of Liver Fibrosis Induced by HCV Genotype 4
Source: Viruses. 2021 Apr 20;13(4):714. doi: 10.3390/v13040714 (PMC8074775; doi:10.3390/v13040714)
Supplement: Supplementary file 1 [file viruses-13-00714-s001.zip › viruses-1139567-supplementary.pdf]

Supplementary Data

Impact of *IL10*, *MTP*, *SOD2*, and *APOE* Gene Polymorphisms on the Severity of Liver Fibrosis Induced by HCV Genotype 4

Amr Ali Hemeda [1], Amal Ahmad Mohamed [2], Ramy Karam Aziz [3], Mohamed S. Abdel-Hakeem [3] and Marwa Ali-Tammam [1,*]

[1] Department of Microbiology and Immunology, Faculty of Pharmaceutical Sciences and Pharmaceutical Industries, Future University in Egypt, Cairo 11835, Egypt; amr.ali@fue.edu.eg

[2] Department of Biochemistry and Molecular Biology, National Hepatology and Tropical Medicine Research Institute, Cairo 11562, Egypt; amalahmedhc@yahoo.com

[3] Department of Microbiology and Immunology, Faculty of Pharmacy, Cairo University, Cairo 11562, Egypt; ramy.aziz@gmail.com (R.K.A.); mohamed.salaheldin@pharma.cu.edu.eg (M.S.A.-H.)

* Correspondence: marwa.ali@fue.edu.eg

Supplementary Table 1: Patients' demographic data

**Demographic data**	**Mild group** (N= 59)	**Severe group** (N= 41)	**Test of Sig.**	***p*-value**
	No.	No.		
**Gender**				
Males	37	29	Chi-square test	0.405
Females	22	12		
**Age (years)**				
Mean ± SD.	41.0 ± 8.0	38.95 ± 7.82	Student t-test	0.207

p-value for comparisons between mild and severe fibrosis groups.

Supplementary Table 2: Patients' clinical data

Laboratory investigations	Mild group (N= 59)	Severe group (N= 41)	Chi-Square *p*-value
	Mean ± SD.	Mean ± SD.	
**Albumin** (g/dL)	3.96 ± 0.37	3.74 ± 0.42	0.008**
**Alpha Feto-Protein (ng/ml)**	13.05 ± 5.87	14.07 ± 18.34	0.033*
**TSH** (IU/L)	3.70 ± 0.71	3.34 ± 0.82	0.022*
**ALP** (IU/L)	136.44 ± 42.97	113.61 ± 53.61	0.037*
**AST** (IU/L)	86.63 ± 60.22	92.29 ± 53.72	0.334
**ALT** (IU/L)	75.85 ± 48.26	68.07 ± 33.47	0.725
**Total Bilirubin** (mg/dL)	1.07 ± 0.37	0.99 ± 0.46	0.125
**Direct bilirubin** (mg/dL)	0.24 ± 0.14	0.27 ± 0.22	0.478
**Blood glucose** (mg/dL)	98.92 ± 16.02	97.46 ± 16.43	0.660
**Hemoglobin (Hb)** (g/dL)	11.91 ± 1.75	11.55 ± 1.80	0.318
**Creatinine (**mg/dL)	0.97 ± 0.16	1.0 ± 0.19	0.847
**Platelet (10^3/ µL))**	271.08 ± 69.61	286.41 ± 75.42	0.450
**WBCs (cells/mm3)**	7938.59 ± 1696.06	7582.90 ± 1803.64	0.150

Chi-square *p*-values for comparisons between mild and Severe groups:
* $p < 0.05$, ** $p < 0.01$, and *** $p < 0.001$.
